# Characterizing the relationship between temperature and mortality in tropical and subtropical cities: a distributed lag non-linear model analysis in Hue, Viet Nam, 2009–2013

**DOI:** 10.3402/gha.v9.28738

**Published:** 2016-01-13

**Authors:** Tran Ngoc Dang, Xerxes T. Seposo, Nguyen Huu Chau Duc, Tran Binh Thang, Do Dang An, Lai Thi Minh Hang, Tran Thanh Long, Bui Thi Hong Loan, Yasushi Honda

**Affiliations:** 1 Department of Health Care Policy and Management, Graduate School of Comprehensive Human Sciences, University of Tsukuba, Tsukuba, Japan; 2 Department of Environmental Health, Faculty of Public Health, University of Medicine and Pharmacy, Ho Chi Minh City, Viet Nam; 3 Department of Global Health Promotion, Graduate School of Medical and Dental Sciences, Tokyo Medical and Dental University, Tokyo, Japan; 4 Department of Pediatrics, Hue University of Medicine and Pharmacy, Hue City, Viet Nam; 5 Institute for Community Heath Research, Hue University of Medicine and Pharmacy, Hue City, Viet Nam; 6 Department of International Cooperation, Ministry of Health, Hanoi, Viet Nam; 7 Department of Epidemiology and Preventive Medicine, Graduate School of Medical and Dental Sciences, University of Kagoshima, Kagoshima, Japan; 8 Department of Global Public Health, Graduate School of Comprehensive Human Sciences, University of Tsukuba, Tsukuba, Japan; 9 Faculty of Health and Sport Sciences, University of Tsukuba, Tsukuba, Japan

**Keywords:** high temperature effects, low temperature effects, hot effects, cold effects, time-series regression

## Abstract

**Background:**

The relationship between temperature and mortality has been found to be U-, V-, or J-shaped in developed temperate countries; however, in developing tropical/subtropical cities, it remains unclear.

**Objectives:**

Our goal was to investigate the relationship between temperature and mortality in Hue, a subtropical city in Viet Nam.

**Design:**

We collected daily mortality data from the Vietnamese A6 mortality reporting system for 6,214 deceased persons between 2009 and 2013. A distributed lag non-linear model was used to examine the temperature effects on all-cause and cause-specific mortality by assuming negative binomial distribution for count data. We developed an objective-oriented model selection with four steps following the Akaike information criterion (AIC) rule (i.e. a smaller AIC value indicates a better model).

**Results:**

High temperature-related mortality was more strongly associated with short lags, whereas low temperature-related mortality was more strongly associated with long lags. The low temperatures increased risk in all-category mortality compared to high temperatures. We observed elevated temperature-mortality risk in vulnerable groups: elderly people (high temperature effect, relative risk [RR]=1.42, 95% confidence interval [CI]=1.11–1.83; low temperature effect, RR=2.0, 95% CI=1.13–3.52), females (low temperature effect, RR=2.19, 95% CI=1.14–4.21), people with respiratory disease (high temperature effect, RR=2.45, 95% CI=0.91–6.63), and those with cardiovascular disease (high temperature effect, RR=1.6, 95% CI=1.15–2.22; low temperature effect, RR=1.99, 95% CI=0.92–4.28).

**Conclusions:**

In Hue, the temperature significantly increased the risk of mortality, especially in vulnerable groups (i.e. elderly, female, people with respiratory and cardiovascular diseases). These findings may provide a foundation for developing adequate policies to address the effects of temperature on health in Hue City.

## Introduction

Climate change is a significant and emerging threat to public health in many countries worldwide, which directly relates to a short-term increase in mortality rates during exposure to low or high temperature (1). Most studies exploring the temperature–mortality relationship have been conducted in developed countries (i.e. North America, Europe), of which the majority are located in temperate and cold climate regions (2). Meanwhile, very few studies have been performed in tropical or subtropical developing countries (3, 4).

According to the Global Climate Index 2015, Viet Nam ranked seventh among the 10 countries most affected by climate change (5). Unsurprisingly, 9 out of 10 of those countries were developing countries, and one country was a middle-income country. In a recent study by Guo et al. (6) that assessed the global variation of high temperature and low temperature effects on mortality, a data set was collected and analyzed from 306 communities in 12 countries (Australia, Brazil, Thailand, China, Taiwan, Korea, Japan, Italy, Spain, the United Kingdom, the United States, and Canada); however, none of the 10 countries most affected by climate change mentioned above were included. This fact may cause an imbalance in assessing the impact of climate change on health.

In temperate and cold climate regions, the temperature–mortality relationship has been confirmed to have the usual U, V, or J shapes (7, 8). However, the latest multicountry study showed an unusual so-called L pattern with a 0–21 lag period, where low temperature effects had a steeper slope and high temperature effects were almost flat (9). Interestingly, these patterns only occurred in tropical or subtropical cities (see Supplementary File 1). The reason for the L pattern being a characteristic in these tropical or subtropical cities remains unclear. In addition, some studies found that both high and low temperature effects resulted in immediate increases in mortality in tropical and subtropical climate areas (10, 11). By contrast, other studies have observed low temperature effects being delayed for several days to weeks in temperate and cold climate areas (7).

A better understanding of the temperature–mortality relationship in tropical or subtropical developing cities is crucial for the establishment of local intervention strategies against temperature effects; it will contribute to projection studies on a global scale (12). We therefore undertook time-series analyses coupled with a distributed lag non-linear model (DLNM) to investigate the short-term (day-to-day variation) association between temperature and mortality in Hue, a subtropical city in Viet Nam. This is the first study in the field using daily mortality data in Viet Nam.

## Methods

### Study area

Viet Nam is located between 8° and 24° north of the equator, having remarkably different climates from the northern to the southern regions. According to the Köppen-Geiger classification, the climate of Southern Viet Nam (e.g. Ho Chi Minh City) can be classified as a ‘tropical wet and dry climate’ (Aw) with an annual mean temperature above 18°C and a dry winter. By contrast, the northern parts (e.g. Hanoi) have a ‘humid subtropical’ (Cwa) climate, with the warmest month over 22°C, the coldest month between −3°C and 18°C, and a dry winter (13). Hue is the capital city of Thua Thien-Hue Province in north-central Viet Nam, 71.7 km^2^ in area and with a population of around 348,000 in 2013 (14). The climate of Hue is considered a ‘tropical monsoon climate’ (Am) under the Köppen-Geiger classification (13). Hue has a mild cold-wet winter and hot-dry summer, with a rainy season from September to January and a dry season from March to August. The yearly average temperature is around 25°C, and the yearly rainfall is approximately 3,000 mm.

### Mortality and weather data

Since 1956, mortality data in Viet Nam has been collected from the civil registration and vital statistics system. The quality of mortality data in this system, however, was very poor; the number of deaths especially was often incomplete and the cause of death inaccurate (15). In 1992, a mortality data-collecting system based on the commune health center was introduced in an official book known as the *A6* (16). Data from the A6 are collected at the commune health center level and then forwarded to the provincial and central levels. The quality of A6 mortality data is adequate, as validated in a previous study (17). In this study, daily mortality data from 27 community health centers in Hue were collected from the A6 mortality reporting system, for the period 2009–2013. The data included information on 6,214 deceased persons with date of death, sex, age, and cause of death classified by the 10th Revision of the International Classification of Disease (ICD10) code. The deceased person was, however, anonymous (only name abbreviations were used). We obtained permission from the Thua Thien-Hue provincial health department before collecting the data. Weather data were obtained from the US National Oceanic and Atmospheric Administration's National Climate Data Center. The necessary information included daily minimum, average, and maximum temperatures, dew point, and relative humidity (18). We did not include air pollution levels in our model due to data unavailability. However, some studies found that temperature effect was not confounded or modified due to air pollution exposure (19–22).

### Statistical model

We used a negative binomial coupled with a DLNM to examine the short-term association (day-to-day variation) between temperature and all-cause mortality (i.e. the daily total number of death counts). Negative binomial distribution was employed to adjust for the Poisson over-dispersion of daily death count *Y*_*t*_ (23). In addition, DLNM was applied to describe the non-linear effect of temperature (in the temperature-mortality dimension) and lag (in the lag-mortality dimension) simultaneously (24). The general model is specified as follows:

1 $$\begin{array}{c} Y_{t}\sim \mathrm{Negative}\mathrm{binomial}\left(\mu_{t}\right)\\ \mathrm{Log}\left(Y_{t}\right)=\alpha +\beta_{l}*T_{t,1}+\beta_{2}*{\mathrm{DOW}}_{t}+\beta_{3}*\mathrm{NCS}\left(\mathrm{time},\mathrm{df}=i/\mathrm{year}\right)+\beta_{4}*\mathrm{NCS}\left(\mathrm{relativehumidity},\mathrm{df}=3\right)+\beta_{5}*\mathrm{NCS}\left(\mathrm{dewpointtemperature},\mathrm{df}=3\right) \end{array}$$

where α is the intercept; *t* is the day of the observation; *Y*_*t*_ is the daily all-cause death count on day *t*; *T*_*t,l*_ is a matrix obtained by applying the ‘cross-basis’ DLNM functions to temperature, β_1_ is the vector of coefficients for *T*_*t,l*_; and *l* is the lag days. Based on previous studies, the natural cubic spline (NCS) with 3 degrees of freedom (df) was selected to control for potential confounding factors (i.e. daily average relative humidity and daily average dew point temperature) (10, 25). *Time* is a continuous variable ranging from 1 on the starting day of observation to 1,811 on the final day of observation within 5 years of data (2009–2013). To adjust for the long-term trend and seasonality, we used NCS smoothing for the time variable with *i* degrees of freedom per year. The day of the week on day *t* (*DOW*_*t*_) was used to control for the effect of day of the week on daily mortality (e.g. on the weekends, mortality tended to be higher than on weekdays). After a series of steps for model selection (Supplementary File 2), the final model of temperature and all-cause mortality included 5 df per year of time variable (*i* value) to control for seasonality and long-term trends and an ‘NCS–NCS’ DLNM using 4 df for the temperature dimension and 5 df for the lag dimension with the maximum lag equal to 28. The model checking procedure was carried out to check the fitness of this final model; it can be found in Supplementary File 3. For the cause-, age-, and sex-specific analyses, the outcome variable, the all-cause daily death count *Y_t_*, was changed to the cause-, age-, and sex-specific daily death count, whereas the structure of predictors was the same as in the final model of all-cause mortality analysis. The cause-specific analysis included four categories: non-external (ICD10 code A00-R99), cardiovascular (ICD10 code I00-I99), respiratory (ICD10 code J00-J99) and cancer mortality (ICD10 code C00-D48). External mortality was excluded due to the very small number of deaths per day (0.2 daily mean). The age-specific analysis included two groups: 0–64 years old and ≥65 years old (the 0–14-year-old group was not separated due to the small number of daily deaths). Given the technical nature of the statistical model, we invite readers to refer to a previous publication by Bhaskaran et al. (26).

### Definition of high and low temperature effects

To quantify the effects of temperature on mortality, we calculated the relative risk (RR) of the low temperature effect, comparing the 1st temperature percentile (15.8°C) to the 50th temperature percentile (26.3°C), and the RR of the high temperature effect, comparing the 99th temperature percentile (32.4°C) to the 50th temperature percentile, using the final DLNM model. RRs can be calculated at single lag (from lag 0 to lag 28), or can be can be calculated at cumulative lag (lag 0–2 for high temperature effect, and lag 0–28 for low temperature effect). For example, the cumulative RR of the high temperature effect on mortality at lag 0–2 is estimated by exp((β_0_+β_1_+ β_2_)*(32.4 − 26.3)), where β_*i*_ are obtained by using a DLNM function of the average temperature with *i*=0, 1, 2 previous days.

## Results

### Descriptive statistics

**Fig. 1:**
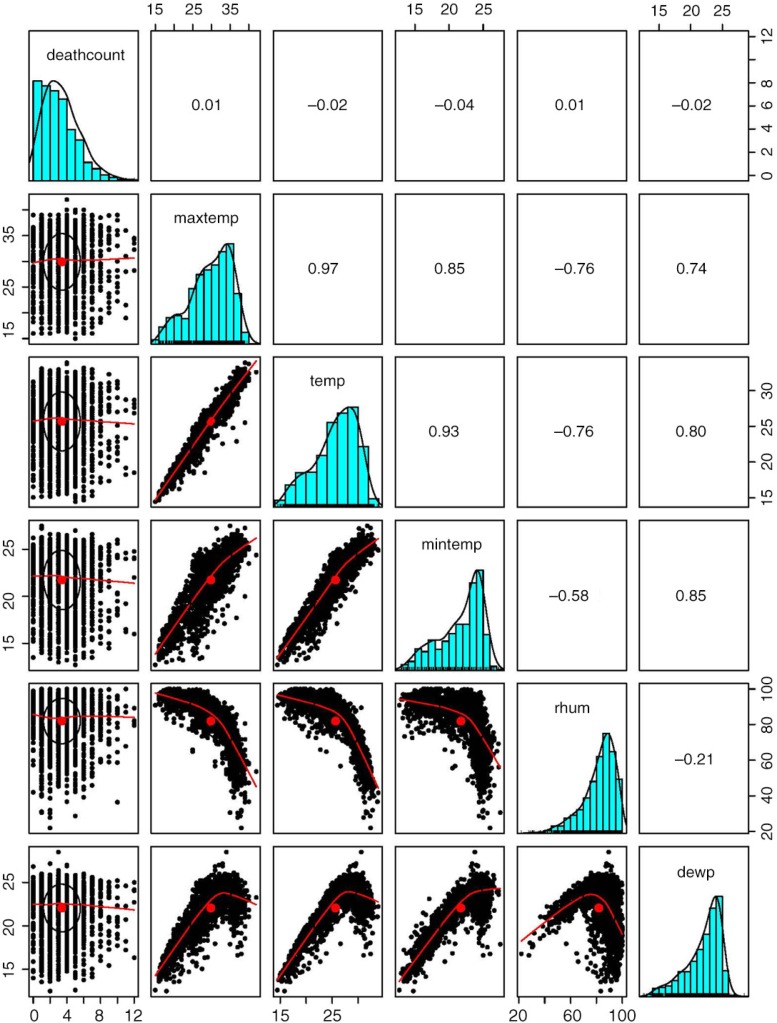
Histograms, scatter plots, and correlation coefficients between weather conditions and mortality in Hue, Viet Nam, 2009–2013.

A total of 6,214 all-cause deaths were recorded in the study period from 2009 to 2013, including 2,215 (35.64%) from cardiovascular diseases and 1,074 (17.28%) from cancer. The other main causes of death in the data were classified as malaise (ICD10 code R53) and cachexia (ICD10 code R64), which amounted to 1,767 cases (accounting for 28.4% of all-cause deaths). These causes of death are, however, mainly associated with aging. We decided to not examine the association between these specific causes and temperature, because we have already included the association analysis between age-specific mortality and temperature as specified in the statistical model section. The proportion of male deaths was slightly higher compared to that of female deaths (53.49% vs. 46.51%). The majority of the deceased were older than 65 years (65.5%). Table 1 shows the descriptive statistics of daily mortality and daily weather conditions. On average, all-cause daily deaths amounted to three cases and ranged from zero to twelve cases. The mean daily maximum temperature was 29.9°C, average temperature 25.7°C, and minimum temperature 21.7°C. These three temperature indicators were strongly associated with each other as shown in Fig. 1.

**Table 1:** Summary statistics of daily weather conditions and daily mortality in Hue, Viet Nam, 2009–2013

				Percentile			
					
Variables	Mean	SD	Minimum	25%	50%	75%	Maximum
Maximum temperature (°C)	29.9	5.5	15	26.2	31	34.2	42
Average temperature (°C)	25.7	4.1	14.4	23	26.3	28.9	33.9
Minimum temperature (°C)	21.7	3.1	12.7	19.8	22.8	24.2	27.5
Average dew point temperature (°C)	22.1	2.7	12.5	20.6	22.9	24.1	28.5
Average relative humidity (%)	81.9	12.7	21.9	75.8	85	91.1	100
All-cause mortality^a^	3.4	2.2	0	2	3	5	12
Cause-specific mortality^a^							
External cause	0.2	0.4	0	0	0	0	4
Non-external cause	3.2	2.1	0	2	3	4	12
Cardiovascular	1.2	1.2	0	0	1	2	7
Respiratory	0.1	0.3	0	0	0	0	2
Cancer	0.6	0.8	0	0	0	1	5
Sex-specific mortality^a^							
Male	1.8	1.5	0	1	2	3	10
Female	1.6	1.4	0	1	1	2	8
Age-specific mortality^a^							
0–14 years old	0.1	0.25	0	0	0	0	2
15–64 years old	1.1	1.1	0	0	1	2	7
≥65 years old	2.2	1.7	0	1	2	3	12

a Mortality is given in number of deaths per day.

### Temperature–mortality relationship

**Fig. 2:**
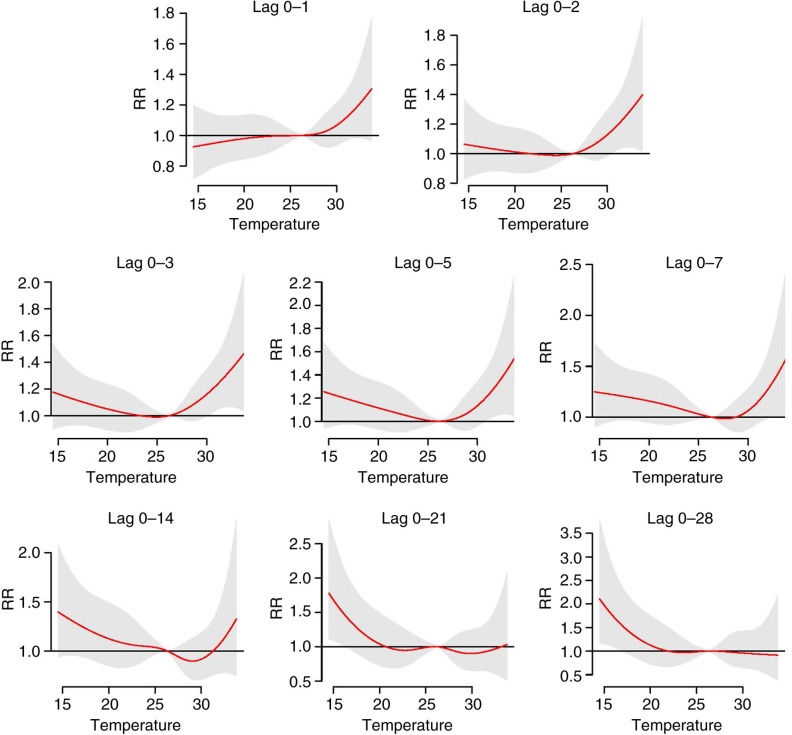
Cumulative overall temperature effects on all-cause mortality at different lag periods. The final natural cubic spline–natural cubic spline (NCS–NCS) model defined by distributed lag non-linear model cross-basis functions with 4 degrees of freedom (df) for the temperature dimension and 5 df for the lag dimension. The reference was at the median of temperature. Red lines are the cumulative relative risks, and grey regions are 95% confidence intervals.

The cumulative overall temperature effects on all-cause mortality at different lag periods are shown in Fig. 2. In lags 0–1 and 0–2, the temperature–mortality relationship had a J-shaped pattern where only high temperatures increased the risks of mortality. In lags 0–3, 0–4 and 0–7, the relationship appeared U-shaped wherein both high and low temperatures increased the risks of mortality. From lag 0–14 to lag 0–28, however, the pattern was L-shaped, wherein only low temperatures significantly increased the risks. These results indicated that the high temperature-related mortality was more associated with short lags, whereas low temperature-related mortality was more associated with long lags.

**Fig. 3:**
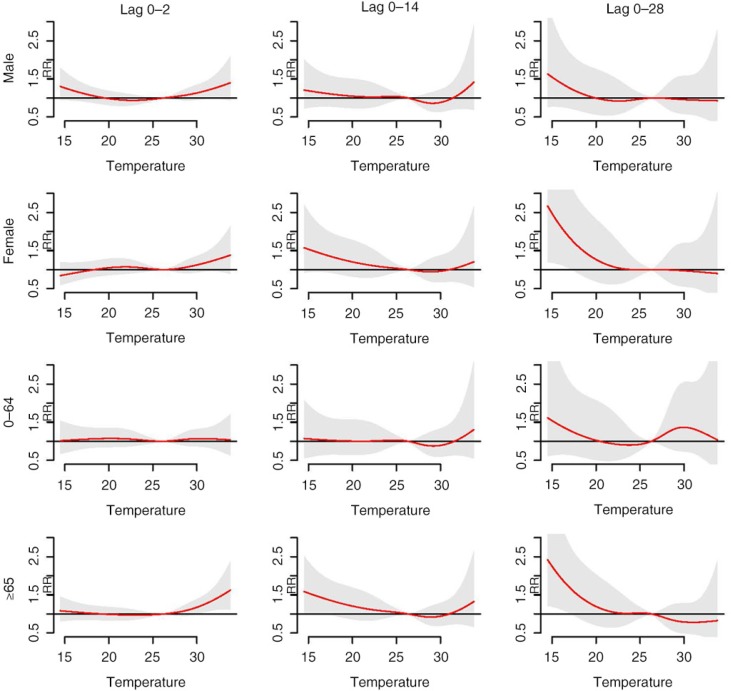
Cumulative overall temperature effects on age- and sex-specific mortality. Red lines are the cumulative relative risks, and grey regions are 95% confidence intervals.

**Fig. 4:**
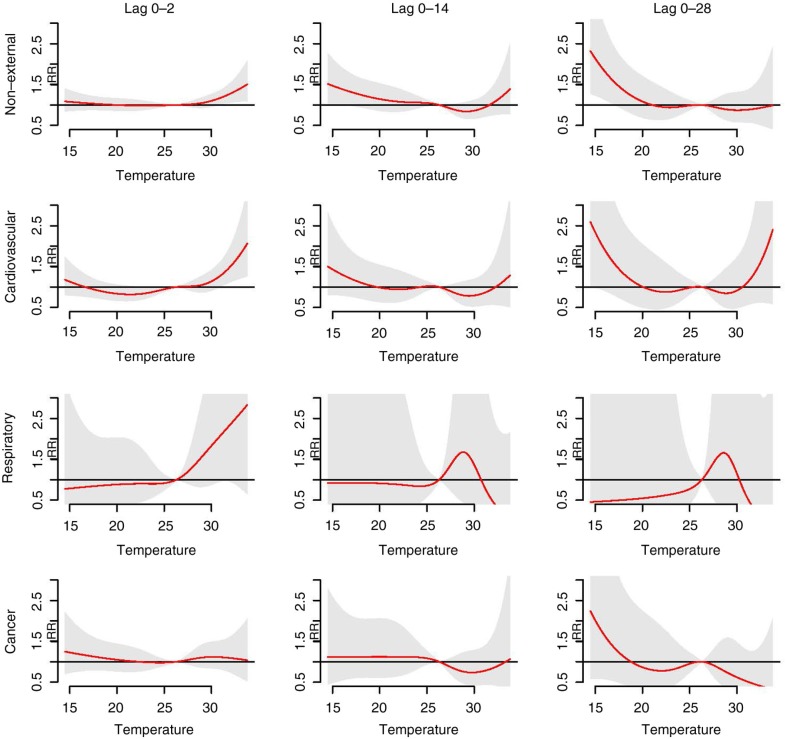
Cumulative overall temperature effects on cause-specific mortality. Red lines are the cumulative relative risks, and grey regions are 95% confidence intervals.

Figure 3 shows the cumulative overall temperature effects on age- and sex-specific mortality. There was no separate analysis for the 0–14 age group due to the small number of daily deaths. The elderly group (≥65 years old) displayed a higher risk of mortality at both high and low temperatures compared to the 0–64 age group. The high temperature effects in short lags (lag 0–2) were similar between male and female. In contrast, the low temperature effects in long lags (lag 0–28) were more prominent among females compared to males. In the cause-specific analysis (Fig. 4), we observed a similar pattern with that of all-cause analysis, wherein high temperature effects were observed in short lags and low temperature effects in long lags, respectively. The exception, however, was cardiovascular mortality, where the high temperature effects manifested in short lags and lasted in long lags. The pattern of temperature mortality in respiratory-related case at long lags was not clear. One of the possible explanations for that is the number of respiratory deaths per day is insufficient.

**Fig. 5:**
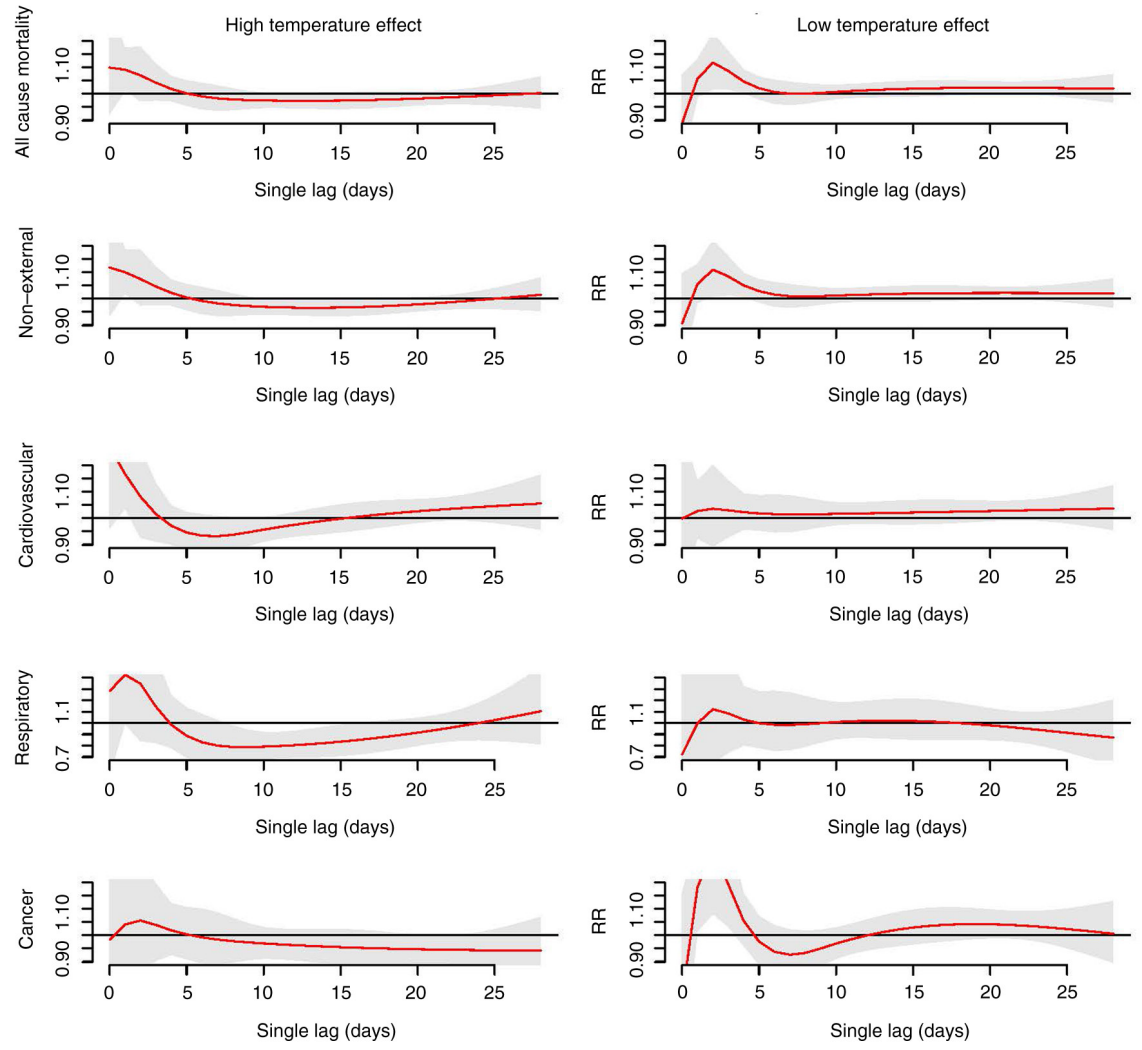
The lag structures of high and low temperature effects on all-cause and cause-specific mortality. The high temperature effect (left) is the effect of the 99th temperature percentile (32.4°C) relative to the 50th temperature percentile (26.3°C). The low temperature effect is the effect of the 1st temperature percentile (15.8°C) relative to the 50th temperature percentile (26.3°C). Red lines are the relative risks at single lag, and grey regions are 95% confidence intervals.

Figure 5 displays high and low temperature effects on all-cause and cause-specific mortality at single lag (please refer to the subsection ‘Definition of high and low temperature effects’ in the Methods for more detail). Both high and low temperature effects caused an immediate increase in the risk of all-cause mortality as well as cause-specific mortality, with high temperatures being affected more acutely than low temperatures (high temperature effects occurred on day 0 vs. low temperature effects, which occurred after 2 days). In addition, high temperatures induced mortality displacement, whereas low temperatures did not show mortality displacement (except for cancer mortality where low temperatures also induced mortality displacement).

Table 2 shows the cumulative RRs of the high temperature effect in lag 0–2 and the low temperature effect in lag 0–28 in cause-, age-, and sex-specific mortality. In all-category mortality (i.e. including cause-, age-, and sex-specific mortality), the RRs of the low temperature effect were higher than the RRs of the high temperature effect (except for respiratory disease). We observed elevated temperature-mortality risk in vulnerable groups: elderly people (high temperature effect, RR=1.42, 95% confidence interval [CI]=1.11–1.83; low temperature effect, RR=2.0, 95% CI=1.13–3.52), females (low temperature effect, RR=2.19, 95% CI=1.14–4.21), people with respiratory disease (high temperature effect, RR=2.45, 95% CI=0.91–6.63), and those with cardiovascular disease (high temperature effect, RR=1.6, 95% CI=1.15–2.22; low temperature effect, RR=1.99, 95% CI=0.92–4.28).

**Table 2:** The cumulative effects of high and low temperatures on cause-, age-, and sex-specific mortality

Statistic	High temperature effect^a^ (95% CI)	Low temperature effect^b^ (95% CI)
All-cause mortality	1.28 (1.04–1.58)*	1.78 (1.10–2.88)*
Cause-specific mortality		
Non-external	1.32 (1.07–1.63)*	1.88 (1.15–3.07)*
Cardiovascular	1.6 (1.15–2.22)*	1.99 (0.92–4.28)
Respiratory	2.45 (0.91–6.63)	0.47 (0.03–8.19)
Cancer	1.08 (0.69–1.68)	1.71 (0.58–5.05)
Sex-specific mortality		
Male	1.28 (0.99–1.67)	1.42 (0.77–2.63)
Female	1.27 (0.95–1.7)	2.19 (1.14–4.21)*
Age-specific mortality		
0–64 years old	1.05 (0.76–1.46)	1.43 (0.65–3.14)
≥65 years old	1.42 (1.11–1.83)*	2.0 (1.13–3.52)*

a High temperature effect is the cumulative relative risk comparing the 99th temperature percentile (32.4°C) to the 50th temperature percentile (26.3°C) at lag 0–2.

b Low temperature effect is the cumulative relative risk comparing the 1st temperature percentile (15.8°C) to the 50th temperature percentile (26.3°C) at lag 0–28.

* Significant at *p*<0.05.

## Discussion

The study examined the temperature–mortality relationship in Hue, Viet Nam, during the period 2009–2013. We found that the temperature–mortality cumulative overall curves changed through lag periods (Fig. 2). For short lags, only high temperature effects were significant (forming a J shape). However, for long lags, only low temperature effects were significant (forming an L shape). McMichael et al. (4) and Wu et al. (27) found that the same phenomenon happened in other tropical and subtropical cities. This phenomenon raised an important issue with regard to choosing the adequate lag periods for modeling the temperature–mortality relationship. For example, most studies chose lag 0–1 to model high temperature effects on mortality, and the authors found significant effects of high temperature on mortality (28). By restricting the study to short lags for high temperatures, however, other characteristics of high temperatures in long lags, such as mortality displacement, may not be fully described.

The cumulative effects of temperature on all-cause mortality had an L shape in lags 0–14, 0–21, and 0–28 (Fig. 2), which was induced by mortality displacement occurring in high temperatures (Fig. 5). Mortality displacement refers to a phenomenon whereby excess daily deaths result from short-term displacement of the time of death (e.g. occurring in most frail individuals whose deaths have only been brought forward by a few days) (29). Another study showed an L-shaped temperature–mortality relationship when quantifying the effect of temperature on mortality in Hanoi (30). Hanoi is in the northeast of Viet Nam and has a similar tropical climate and temperature distribution to Hue. The study in Hanoi, however, used monthly data; therefore the occurrence of mortality displacement could not be fully examined. In addition, other studies using daily mortality data in tropical/subtropical regions also showed an L-shaped pattern (27, 31). The lag structures of these studies, nevertheless, were not described in detail to confirm whether or not mortality displacement occurred. Basu et al. (32) and Hajat et al. (33) addressed the presence or absence of mortality displacement depending on several factors including the baseline health status of the population (presence of chronic diseases), the population at risk (elderly people), and other local factors. Mortality displacement occurring in Hue is understandable, because a majority of deaths were attributed to chronic diseases (35.64% to cardiovascular disease and 17.28% to cancer) and the proportion of deaths among those older than 65 years was quite high (65.5% of the total deaths).

Previous studies tried to project the impact of heat-related death on a global scale (34). As pointed out by Honda et al. (12) the estimation of optimum temperature (OT) and the risk function of temperature on mortality in each area were needed to conduct the projection on a global scale. To estimate the OT, the temperature mortality had to be assumed to have a V shape (where the OT is the base of the V shape). In Hue, however, and in other tropical/subtropical cities (as shown in Supplementary File 1), the temperature–mortality relationship had an L shape with long lags. Therefore, the spatial pattern of temperature mortality should also be taken into account when projecting the impact of heat-related death on a global scale. Hajat et al. (2) and Seposo et al. (35) showed the paucity of research on the effect of temperature on mortality in tropical/subtropical developing areas compared to temperate/cold developed areas. Thus, in order to improve our projection of heat-related deaths on a global scale, more studies from tropical/subtropical developing areas are warranted.

Hue, a subtropical city in Viet Nam showed higher mortality risk induced by low temperatures (in long lags) compared to high temperatures (in short lags) in all-category mortality (Table 2). Other studies in subtropical regions (i.e. Brisbane, Australia, and Guangzhou, China) reported that mortality in winter was higher than in summer (36, 37). In a multicountry study, Gasparrini et al. (9) found that the attributable deaths were more pronounced for low than for high temperatures, and the differences in attributable deaths between low and high temperatures were even greater in tropical or subtropical cities (see Supplementary File 1). These results suggest that populations in subtropical regions suffer more from low temperature effects than high temperature effects. Within the context of global warming, many previous studies focused on the high temperature effects rather than low temperature effects. However, this finding indicates that the government of Hue City should pay attention to both high and low temperature effects when developing health policies in order to reduce the impact of temperature effects. In addition, the acute low temperature effects in this study (Fig. 5) were also observed in other tropical/subtropical regions such as in the city of Chiang Mai, Thailand (Köppen-Geiger tropical wet and dry climate, Aw) (10), Monterey, California (Köppen-Geiger dry summer subtropical climate, Csb), São Paulo, Brazil (Köppen-Geiger humid subtropical climate, Cfa), and Mexico (Köppen-Geiger subtropical highland climate, Cwb) (4). This phenomenon is easily understood because people in tropical and subtropical regions are not well acclimatized to cold weather.

Regarding the age-specific analysis, the effects of both high and low temperature were greater among the elderly (≥65 years old) compared to the 0–64 age group (Fig. 3 and Table 2). Numerous studies have provided similar evidence that the elderly population is among the most vulnerable groups (28, 29, 38). Aging induces a decrease in thermoregulatory abilities, together with the increased prevalence of chronic diseases, which are likely to contribute to vulnerability to temperature effects in elderly people (39). We found that low temperature effects were more pronounced for females than for males, which is in line with a study by Ou et al. (36). The high temperature effects, however, were not significantly different in females compared to males. So far the evidence that sex modifies the effects of high temperature on mortality depends on location and population (29, 40). We also observed that the RR of high temperature was highest in respiratory mortality (Table 2). One of the physiological mechanisms that triggers respiratory deaths induced by high temperatures is that high temperatures can affect the lung function of chronically ill and older people (41, 42). It should be noted that the effects were observed in cardiovascular mortality in both high and low temperature (Fig. 4 and Table 2). This finding implies that patients with cardiovascular disease should be taken care of during both hot and cold periods. Losing water and salt from sweating during exposure to high temperatures can cause hemoconcentration, which in turn leads to thrombosis. Moreover, exposure to low temperatures slows down blood flow to the skin in order to preserve heat and increases blood cholesterol, levels of red blood cell counts, and plasma fibrinogen. It also induces thrombosis due to hemoconcentration (43).

Selecting an appropriate model is crucial when examining temperature effects on mortality, as it can affect the ability to make a prediction (7). In this study we proposed an objective-oriented DLNM approach based on the Akaike information criterion rule in analyzing the temperature–mortality relationship rather than making strong prior assumptions. For example, we chose the df for the time variable to control for seasonality and long-term trends, the best temperature indicators (i.e. maximum, average, or minimum temperature), as well as the best-fit df for NCS–NCS in the temperature and lag dimensions.

Our research had some limitations, such as the lack of control for air pollution. The effect modification of air pollution, however, seems to be negligible; thus its inclusion might not really alter the relationship (19–22). The A6 mortality data information contained some missing values, and the causes of death were misclassified in some cases (i.e. inconsistencies between the cause of death in text and ICD codes). In order to ensure the quality of mortality data, we sent our facilitators to every community health center for random checking and collection of missing values.

## Conclusions

This is the first study using daily all-cause and cause-specific mortality data to examine the effects of temperature on mortality in Hue, Viet Nam. In Hue, high temperature-related mortality was more associated with short lags, whereas low temperature-related mortality was more associated with long lags. Both high and low temperature effects occurred acutely, but low temperature effects lasted longer than high temperature effects and the high temperature effects induced mortality displacement. Low temperatures increased risk in all-category mortality compared to high temperatures. We observed that elderly people, females, and patients with cardiovascular and respiratory disease were the most vulnerable groups affected by temperatures. These findings may provide a foundation for developing adequate policies to address the effects of temperature on health in Hue City.

## Supplementary Material

Characterizing the relationship between temperature and mortality in tropical and subtropical cities: a distributed lag non-linear model analysis in Hue, Viet Nam, 2009–2013
