# Supplementary material for: Characterizing the relationship between temperature and mortality in tropical and subtropical cities: a distributed lag non-linear model analysis in Hue, Viet Nam, 2009–2013
Source: Glob Health Action. 2016 Jan 13;9:10.3402/gha.v9.28738. doi: 10.3402/gha.v9.28738 (PMC4716554; doi:10.3402/gha.v9.28738)
Supplement: Characterizing the relationship between temperature and mortality in tropical and subtropical cities: a distributed lag non-linear model analysis in Hue, Viet Nam, 2009–2013 [file GHA-9-28738-s001.pdf]

Supplemental Material

Characterizing temperature and mortality relationship in tropical and subtropical Cities: A distributed lag nonlinear model analysis in Hue, Viet Nam, 2009-2013

Tran Ngoc Dang, Xerxes T. Seposo, Nguyen Huu Chau Duc, Tran Binh Thang, Do Dang An, Lai Thi Minh Hang, Tran Thanh Long, Bui Thi Hong Loan, Yasushi Honda

Table of contents

Additional file 1

Figure S1. The temperature-mortality relationship and Kȍppen climate classification of some tropical/subtropical cities

Additional file 2

Model selection procedure

Model selection results

Additional file 3: Model checking

Figure S2. Residual deviances plot of the final DLNM model

Figure S3. Sensitivity analysis of the final DLNM model when extending maximum lags up to 45 days.

References

Additional file 1

Figure S1. The temperature-mortality relationship and Kòppen climate classification of some tropical/subtropical cities.

This figure was modified from the paper: Gasparrini A, Guo Y, Hashizume M, Lavigne E, Zanobetti A, Schwartz J, et al. Mortality risk attributable to high and low ambient temperature: a multicountry observational study. Lancet. 2015;386(9991):369-75

The L-shaped temperature-mortality in these tropical/subtropical cities were reproduced from Figure S1 in above mentioned paper.

Name of the city	Country	Climate Kòppen system (1)	Location (lat-long)	Temperature-mortality relationship (2)
Cuiaba	Brazil	Tropical wet and dry climate- Aw	15.5958° S, 56.0969° W	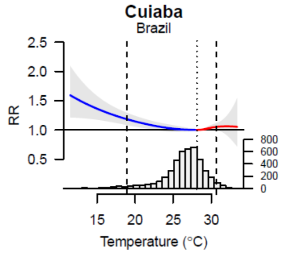
Salvador	Brazil	Tropical rain forest - Af	12.9747° S, 38.4767° W	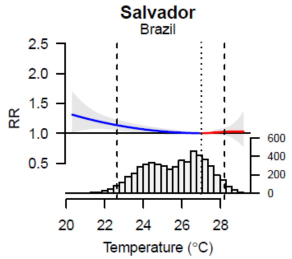

Fukui	Japan	Humid subtropical climate - Cfa	36.0667° N, 136.2167° E	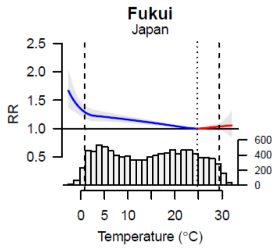
Fukuoka	Japan	Humid subtropical climate - Cfa	33.5833° N, 130.4000° E	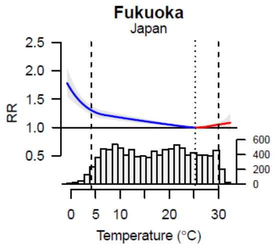
Hyogo	Japan	Humid subtropical climate - Cfa	34.6908° N, 135.1831° E	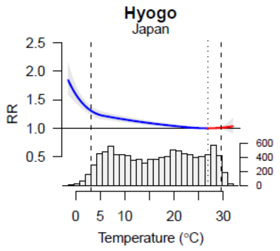
Kochi	Japan	Tropical monsoon climate - Aw	33.5667° N, 133.5333° E	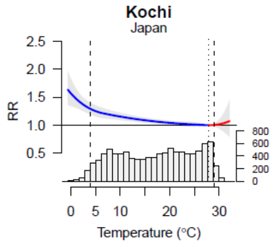

<table>
<tr><td>Kumamoto</td><td>Japan</td><td>Humid subtropical climate - Cfa</td><td>32.7833° N, 130.7333° E</td><td>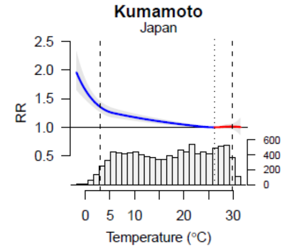
</td></tr>
<tr><td>Okayama</td><td>Japan</td><td>Humid subtropical climate - Cfa</td><td>34.6500° N, 133.9167° E</td><td>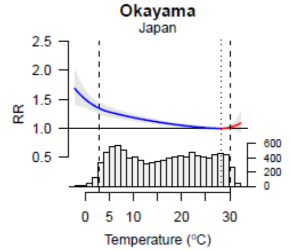
</td></tr>
<tr><td>Okinawa</td><td>Japan</td><td>Tropical rain forest - Af</td><td>26.5000° N, 128.0000° E</td><td>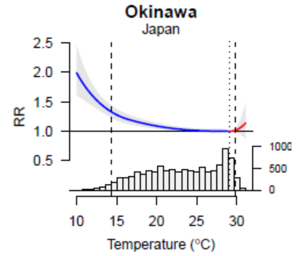
</td></tr>
<tr><td>Saga</td><td>Japan</td><td>Humid subtropical climate - Cfa</td><td>33.2667° N, 130.3000° E</td><td>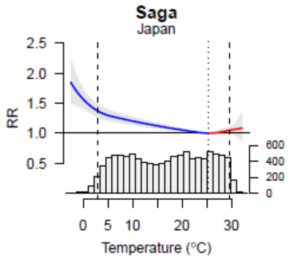
</td></tr>
</table>

Shiga	Japan	Humid subtropical climate - Cfa	35.1167° N, 136.0667° E	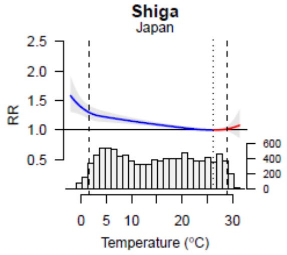
Shimane	Japan	Humid subtropical climate - Cfa	35.2167° N, 132.6667° E	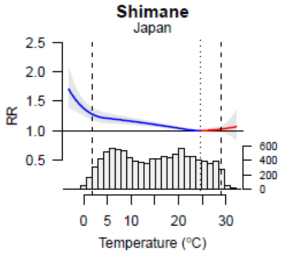
Wakayama	Japan	Humid subtropical climate - Cfa	34.0500° N, 135.3500° E	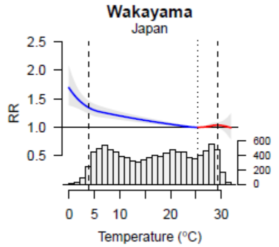
Busan	Korea	Humid subtropical climate - Cwa	35.1794° N, 129.0756° E	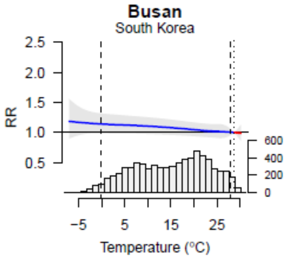

Taichung	Taiwan	Humid subtropical climate - Cwa	24.1500° N, 120.6667° E	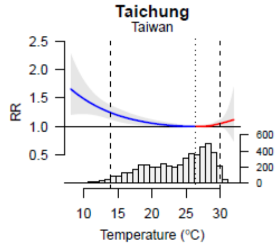
Atlanta, GA	USA	Humid subtropical climate - Cfa	33.7550° N, 84.3900° W	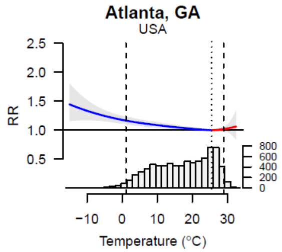
Brownsville, TX	USA	Humid subtropical climate - Cfa	25.9303° N, 97.4844° W	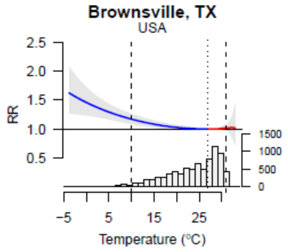
Charlotte, NC	USA	Humid subtropical climate - Cfa	35.2269° N, 80.8433° W	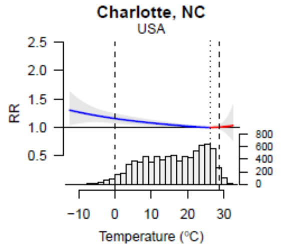

Daytona Beach, FL	USA	Humid subtropical climate - Cfa	29.1900° N, 81.0894° W	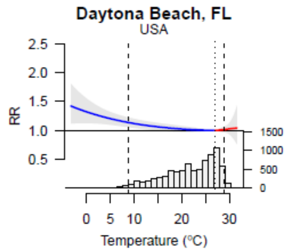
Naples, FL	USA	Tropical wet and dry climate- Aw	26.1500° N, 81.8000° W	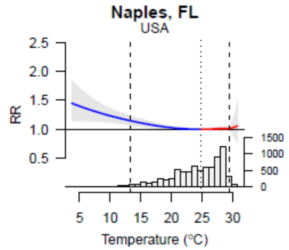
Tulsa, OK	USA	Humid subtropical climate - Cfa	36.1314° N, 95.9372° W	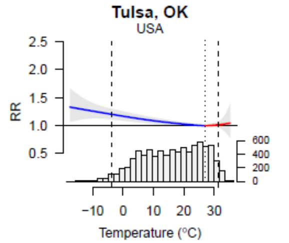

Additional file 2

Model selection procedure

We used a negative binomial coupled with a distributed lag nonlinear model (DLNM) to examine the temperature effect on all-cause mortality. Negative binomial distribution was employed to adjust for the Poisson over-dispersion of daily death count Yt (3). In addition, DLNM was applied to describe the nonlinear effect of temperature (in the temperature-mortality dimension) and lag (in the lag-mortality dimension) simultaneously (4). The general model is specified as follows:

$Y_t \sim Negative\ binomial\ (\mu_t)$

$Log\ (Y_t) = \alpha + \beta_1 * T_{t,l} + \beta_2 * DOW_t + \beta_3 * NCS\ (time,\ df=i/year) + \beta_4 * NCS(relative\ humidity,\ df=3) + \beta_5 * NCS(dewpoint\ temperature,\ df=3)$

where α is the intercept; t is the day of the observation; Y_t is daily all-cause death count on day t; $T_{t,l}$ is a matrix obtained by applying the "cross-basis" DLNM functions to temperature, β_1 is the vector of coefficients for $T_{t,l}$, and l is the lag days. According to previous studies, the natural cubic spline (NCS) with three degrees of freedom (df) was selected to control for potential confounding factors (i.e. daily average relative humidity and daily average dewpoint temperature) (5, 6). *Time* is a continuous variable ranging from 1 on the starting day of observation to 1811 on the final day of observation within five years of data (2009-2013). To adjust for the long-term trend and seasonality, we used NCS smoothing for the *time* variable with *i* degrees of freedom per year. The day of the week on day t (DOW_t) was used to control for the effect of weekday on daily mortality (e.g. on the weekends, mortality tended to be higher than that on week days).

There are varieties of possible models can be chosen in Equation 1 due to the flexible choice of DLNM functions as well as the choice of df for controlling seasonality and long term trend (i.e. i value). In this study, we attempted to propose an objective-oriented procedure for the ultimate model selection through four steps. This selection procedure strictly follows the AIC rule (i.e. a smaller AIC value is the better model) which has been used in environmental epidemiology time series regression analyses (6, 7). The four steps of ultimate model selection are described as below:

Step 1: Goodness of fit for seasonal and long-term trend control.

First, we fitted a simpler model from Equation 1 with the weekday and time variables:

$$Log\ (Y_t) = \alpha + DOW_t + NCS\ (time,\ df = i/year) \quad (2)$$

We then changed the df per year (i.e. *i* value) from 1 to 24 and chose the best fit *i* value based on the smallest AIC value.

Step 2: Determine the best combination of df between the temperature-mortality and lag-mortality dimensions using NCS-NCS cross-basis functions.

First, we held the maximum lag values at 14, 21, and 28 respectively. These values were most frequently used in previous studies (2, 8-10).

Secondly, we applied the cross-basis functions to the temperature variable ($T_{t,l}$), and varied the df of NCS from 4 to 10 in the temperature dimension as well as in the lag dimension. The R code for this purpose is specified below; for more details please refer to the "dlnmTS" vignette in R software:

> $T_{t,l}$ <- crossbasis (temp, lag=14, argvar=list (fun="ns", df=k, cen=median(temp), arglag=list(fun="ns", df=j,logknots(14, j-2)

This R code implies that k is the df of NCS in the temperature dimension; j is the df of NCS in the lag dimension. After that, we updated the Equation 2 with the temperature variable after applying cross-basis functions (i.e., $T_{t,l}$) and the best value of df per year controlling for seasonality and long-term trend, which is shown below:

$$Log\ (Y_t) = \alpha + DOW_t + NCS\ (time,\ df = i/year) + T_{t,l} \quad (3)$$

Step 3: Checking the necessity for controlling relative humidity and dew point temperature.

We updated Equation 3 with relative humidity and dew point temperature and observed how the AIC value changed. If the AIC value is significantly smaller than the smallest AIC of model in Equation 3, then relative humidity and dew point temperature should be controlled. Otherwise, these two variables are excluded.

Step 4: Checking the best temperature indicator.

So far, in Equation 3 we used the average temperature as the temperature indicator. However, we also checked the performance of minimum and maximum temperatures by updating $T_{t,l}$ using these two temperature indicators.

Model selection results:

As described in the model selection procedure above, we performed model selection based on the AIC rule (i.e. a smaller AIC is the better model) and followed the four steps. In Step 1, our analysis showed that the df value per year of time variable equal to 5 (*i* value) was the best value as the control for seasonality and long-term trend. In Step 2, we observed that the maximum lag value equal to 28 with 4 df for the temperature (k value) and 5 df for the lag (j value) were the best values resulting in the smallest AIC at 7533.287. According to the default function of "dlnm" package (4), “k=4” means three internal knots at equally-spaced percentiles in the temperature dimension and “j=5” means three internal knots equally spaced on the log values of lag. In Step 3, the AIC value was 7538.729 when controlling for relative humidity and was 7538.147 when controlling for dew point temperature. These values were higher than the smallest AIC value in Step 2, which indicated that it was not necessary to include relative humidity and dew point temperature in the model. In Step 4, the AIC showed that the average temperature was slightly better compared to minimum and maximum temperatures in the Hue data set. Therefore, we chose to report the average temperature. In summary, the ultimate model in quantifying temperature-mortality relationship was "NCS - NCS" DLNM with 4 df for average temperature dimension and 5 df for lag dimension.

For the purpose of visualization of model selection results, please consult the power point slides below:

Model selection visualization results additional file 2

Tran Ngoc Dang

Nov,30th, 2015

Step 1. goodness of fit for seasonal and long-term trend control

- glm(deathcount ~ ncs(time,df=5*i) + as.factor(dow), negative binomial);
- i varies from 1 to 24

	row.names	V1
1	AIC.season.df 1	7733.560
2	AIC.season.df 2	7690.554
3	AIC.season.df 3	7661.347
4	AIC.season.df 4	7654.918
5	AIC.season.df 5	7645.805
6	AIC.season.df 6	7649.142
7	AIC.season.df 7	7651.028
8	AIC.season.df 8	7648.949
9	AIC.season.df 9	7655.187
10	AIC.season.df 10	7659.387
…		
23	AIC.season.df 23	7694.934
24	AIC.season.df 24	7703.948

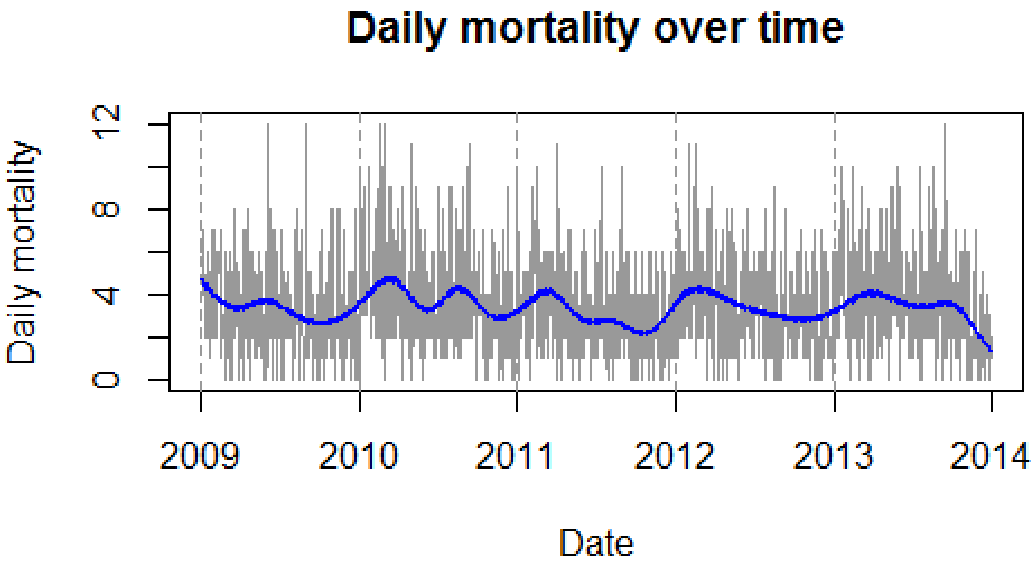

- time is continuous variable ranging from 1 at start day of observation to 1811 at final day of observation within five years data 2009-2013

-dow=day of the week: control the effect of weekday on daily mortality

Step 2. best combination of df between temp and lag

```
argvar=list(fun="ncs", df=i)
arglag=list(fun="ncs",df=j, logknots(21,j-2)))
```

- Maxlag=14

	row.names	lag.df.4	lag.df.5	lag.df.6	lag.df.7	lag.df.8	lag.df.9	lag.df.10
1	temp.df.4	7591.627	7597.448	7599.022	7601.878	7605.791	7612.282	7612.375
2	temp.df.5	7598.760	7606.545	7609.807	7614.437	7620.141	7625.640	7626.044
3	temp.df.6	7601.759	7609.944	7614.822	7621.426	7624.339	7629.620	7630.040
4	temp.df.7	7607.285	7618.341	7624.184	7631.742	7637.004	7644.161	7644.536
5	temp.df.8	7603.759	7614.991	7623.154	7632.724	7638.980	7647.816	7648.229
6	temp.df.9	7607.412	7621.578	7630.879	7639.612	7645.670	7656.768	7657.162
7	temp.df.10	7611.765	7618.601	7631.928	7636.549	7643.421	7656.561	7656.882

- Maxlag=21

	row.names	lag.df.4	lag.df.5	lag.df.6	lag.df.7	lag.df.8	lag.df.9	lag.df.10
1	temp.df.4	7560.700	7563.896	7566.435	7570.629	7576.241	7579.932	7586.067
2	temp.df.5	7568.169	7573.262	7577.778	7583.890	7591.347	7596.771	7602.533
3	temp.df.6	7571.372	7577.364	7582.875	7590.791	7599.820	7603.266	7609.100
4	temp.df.7	7578.190	7583.961	7590.801	7601.157	7611.355	7617.134	7624.871
5	temp.df.8	7573.254	7578.804	7587.219	7598.681	7610.557	7617.484	7626.700
6	temp.df.9	7576.882	7584.993	7595.292	7608.295	7618.736	7626.021	7637.569
7	temp.df.10	7579.367	7587.452	7593.948	7605.590	7617.900	7624.406	7637.525

- Maxlag=28

	row.names	lag.df.4	lag.df.5	lag.df.6	lag.df.7	lag.df.8	lag.df.9	lag.df.10
1	temp.df.4	7533.414	7533.287	7539.656	7543.560	7547.731	7552.727	7557.785
2	temp.df.5	7540.264	7542.376	7550.746	7556.553	7562.666	7569.761	7574.218
3	temp.df.6	7543.599	7547.152	7555.586	7562.885	7570.912	7578.137	7580.460
4	temp.df.7	7550.663	7552.583	7564.282	7573.202	7582.203	7591.219	7596.121
5	temp.df.8	7546.298	7548.736	7561.655	7572.593	7583.665	7594.105	7599.727
6	temp.df.9	7548.344	7552.822	7568.417	7580.359	7590.815	7599.719	7606.642
7	temp.df.10	7551.352	7556.329	7566.366	7581.905	7584.836	7595.767	7604.385

Step 3. necessity for controlling relative humidity and dewpoint temperature

- Model 1=glm(deathcount ~ f(ti|β) + ncs(time,df=5*5) + as.factor(dow), negative binomial)
- f(ti|β) : ncs(temp, df=4) ; ncs(lag, df=5, maxlag=28) – from step 2

model2<-update(model1, .~. + ns(dewp,df=3))

model3<-update(model1, .~. + ns(rhum,df=3))

AIC of model 2= 7538.147

AIC of model 3= 7538.729

AIC of model1 = 7533.287

Step 4. checking temperature indicator

Model 1= glm(deathcount ~ f(ti|β) + ncs(time,df=5*5) + as.factor(dow), negative binomial)

– f(ti|β) : ncs(indicator, df=4) ; ncs(lag, df=5, maxlag=28); varied indicator= average temperature, max temperature, min temperature respectively

	row.names	V1
1	AIC.indicator temp	7533.287
2	AIC.indicator maxtemp	7533.865
3	AIC.indicator mintemp	7536.850

So far the best NCS-NCS model is…

– Model ultimate = glm(deathcount ~ f(ti|β) + ncs(time,df=5*5) + as.factor(dow), negative binomial)
– f(ti|β) : ncs(temp, df=4) ; ncs(lag, df=5, maxlag=28)

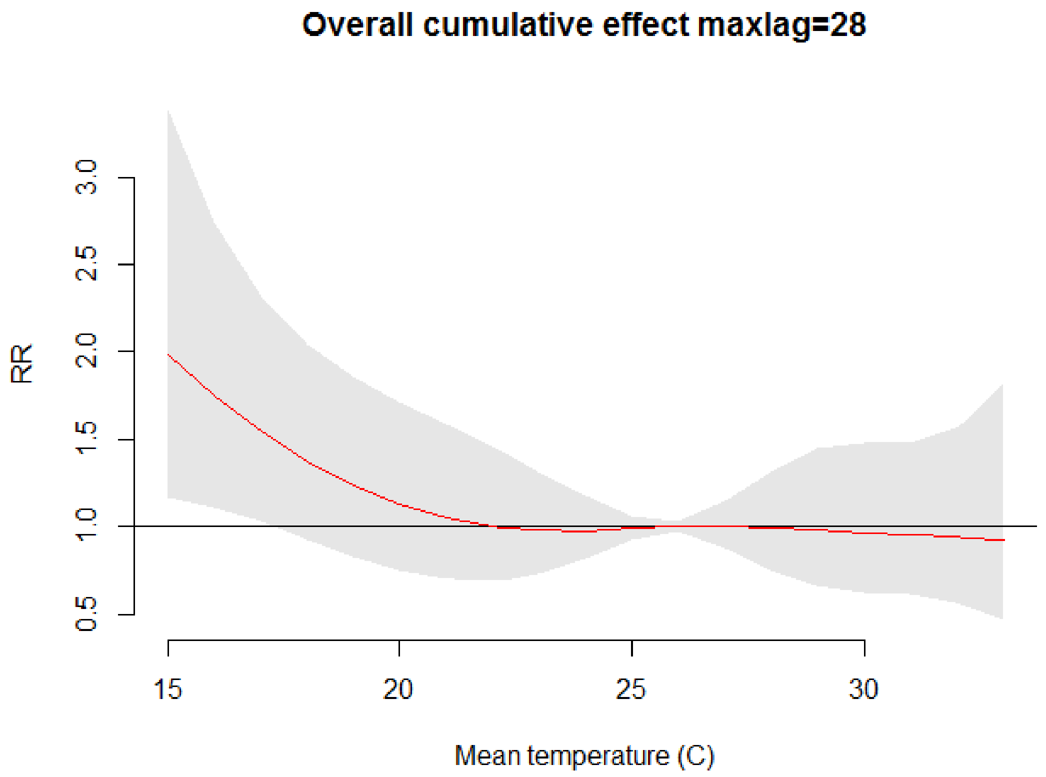

Additional file 3: Model checking

Figure S2. Residual deviances of the final DLNM model

The final DLNM model is NCS with 4df for the dimension of temperature and NCS with 5df for the dimension of lag. Max lag value is 28 days.

Top is Scatter plot of deviance residuals vs time. Below is partial autocorrelation (ACF) plot of the deviance residuals

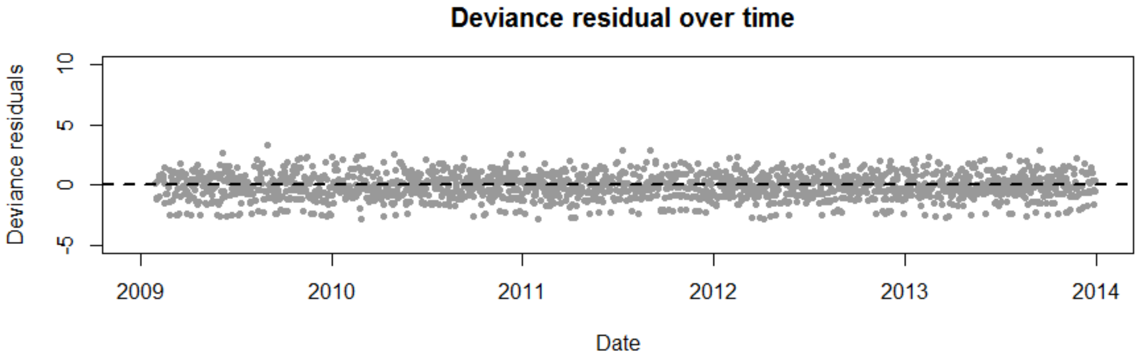

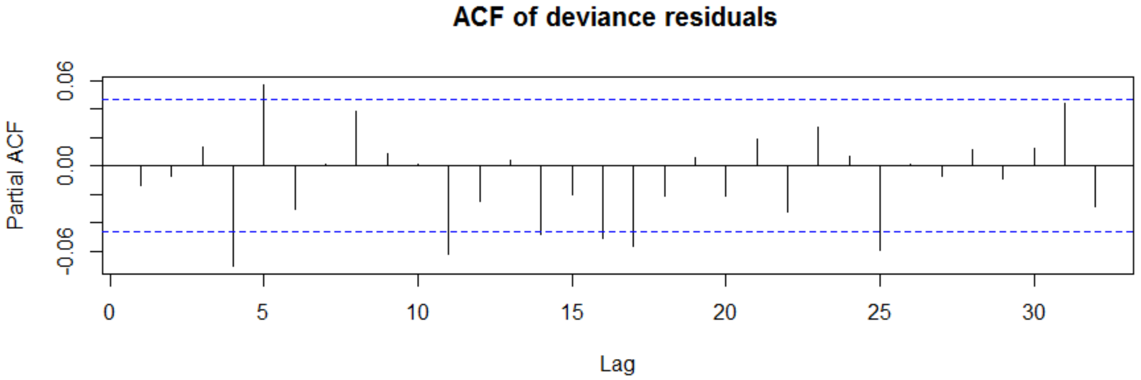

Figure S3. Sensitivity analysis of the final DLNM model when extending maximum lags up to 45 days.

We extended the maximum lag value up to 45 days (the uper limit value allowed in "dlnm" package), and compared the patterns of RR of low temperature effect at single lag between maximum lag = 28 days vs. maximum lag = 45 days. The patterns of RR at single lag between the two maximum lag values were quite similar. In addition, the cumulative RRs at lag 0-28 were 1.78, and 1.81 in maximum lag = 28 days, and maximum lag = 45 days, respectively. This indicated that when lag time increased further, the cumulative RR at lag 0-28 was only slightly increased. It is, however, important to notice that the maximum lag value should not exceed 28 days, because the effects of low temperature have been reported to only last up to three/four weeks (2), and the maximum lag values at 14, 21, and 28 were the most frequently used in literature (8-10). Although we cannot rule out additional effects when extending the lags, our model seems able to capture most of the association

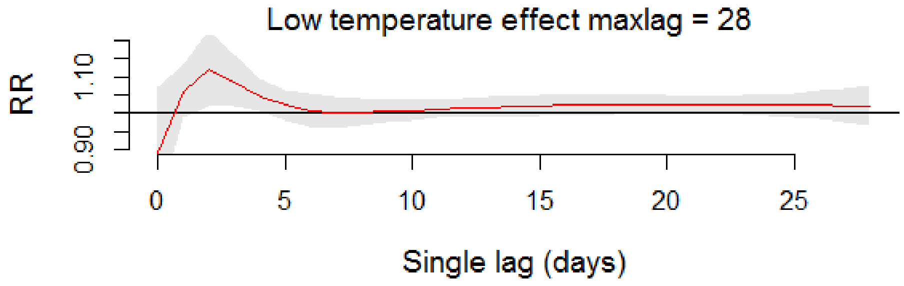

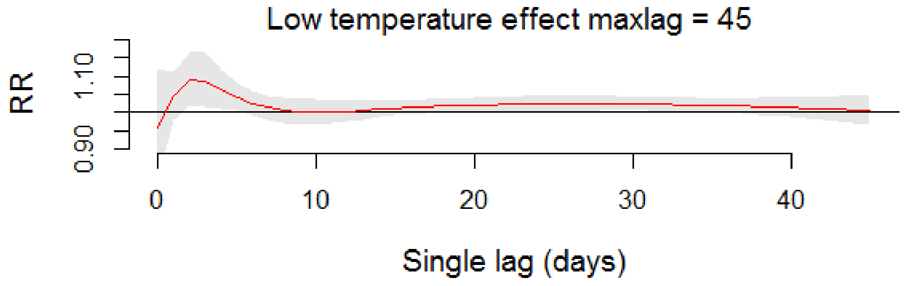

References

1. Peel MC, Finlayson BL, McMahon TA. Updated world map of the Köppen-Geiger climate classification. Hydrol Earth Syst Sci. 2007;11(5):1633-44.

2. Gasparrini A, Guo Y, Hashizume M, Lavigne E, Zanobetti A, Schwartz J, et al. Mortality risk attributable to high and low ambient temperature: a multicountry observational study. Lancet. 2015.

3. Ver Hoef JM, Boveng PL. Quasi-Poisson vs. negative binomial regression: how should we model overdispersed count data? Ecology. 2007;88(11):2766-72.

4. Gasparrini A, Armstrong B, Kenward MG. Distributed lag non-linear models. Statistics in medicine. 2010;29(21):2224-34.

5. Guo Y, Punnasiri K, Tong S. Effects of temperature on mortality in Chiang Mai city, Thailand: a time series study. Environmental health : a global access science source. 2012;11:36.

6. Peng RD, Dominici F, Louis TA. Model choice in time series studies of air pollution and mortality. J Roy Stat Soc a Sta. 2006;169:179-98.

7. Armstrong B. Models for the relationship between ambient temperature and daily mortality. Epidemiology. 2006;17(6):624-31.

8. Guo YM, Gasparrini A, Armstrong B, Li SS, Tawatsupa B, Tobias A, et al. Global Variation in the Effects of Ambient Temperature on Mortality A Systematic Evaluation. Epidemiology. 2014;25(6):781-9.

9. Bai L, Cirendunzhu, Woodward A, Dawa, Xiraoruodeng, Liu Q. Temperature and mortality on the roof of the world: a time-series analysis in three Tibetan counties, China. The Science of the total environment. 2014;485-486:41-8.

10. Wu W, Xiao Y, Li G, Zeng W, Lin H, Rutherford S, et al. Temperature-mortality relationship in four subtropical Chinese cities: a time-series study using a distributed lag non-linear model. The Science of the total environment. 2013;449:355-62.
